# The elusive nature of mucormycosis in an immunocompetent host and the role of a dermatology consult

**DOI:** 10.1002/ccr3.2479

**Published:** 2019-10-02

**Authors:** Dylan J. Badin, Catherine Baker, Brian J. Simmons, Shaofeng Yan, Kathryn A. Zug

**Affiliations:** ^1^ Section of Dermatology Department of Surgery Dartmouth Hitchcock Medical Center Lebanon NH USA; ^2^ Department of Pathology Dartmouth Hitchcock Medical Center Lebanon NH USA

**Keywords:** dermatology, immunocompetent, mucormycosis, rhizopus, trauma

## Abstract

Mucormycosis infection in the immunocompetent host typically occurs in the setting of trauma and presents a diagnostic challenge. The earliest signs of infection are often cutaneous, but can easily be misinterpreted, which can be a fatal mistake. Dermatology has tools to help recognize these infections and initiate earlier therapy.

## Graphical abstract

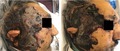

## 1 INTRODUCTION

Mucormycosis is traditionally a pathology of the immunocompromised, but trauma can elicit infection in the immunocompetent. The complexity of these traumatic injuries is often neglected, as is the elusive nature of infection. Here, we highlight the diagnostic challenge following trauma and define the role of a dermatology consultation in recognition.

Angioinvasive mucormycosis is an opportunistic infection that typically afflicts poorly controlled diabetic and/or immunocompromised patients. The traditional means of acquisition is through either inhalation or direct inoculation in the setting of trauma.^1^, ^2^ With reports of in‐hospital mortality from 30% to 50% of infected patients,^2^ early recognition and treatment of the infection is critical. Because mucormycosis is rarely seen in an immunocompetent host, it can easily be missed, leading to delays in treatment. We present a case of invasive mucormycosis infection in an immunocompetent host, highlighting its elusive nature with it initially being mistaken for both post‐op infection and frostbite. We discuss the complex presentation of opportunistic fungal infections and discuss the important role of early dermatologic consultation to facilitate the rapid recognition and treatment.

## 2 CASE

**Figure 1:**
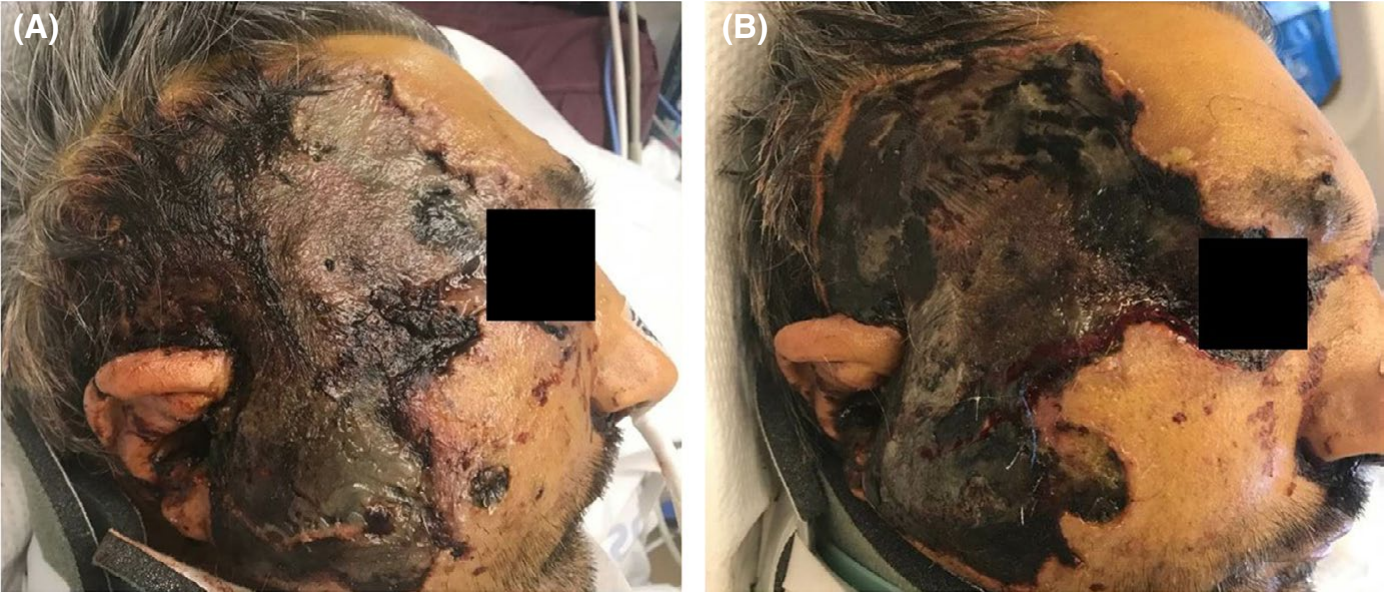
A, Necrotic plaque on right cheek, forehead, and right periocular area from hospital day 22. B, Rapid expansion of plaque over 24 h with increased areas of skin necrosis

**Figure 2:**
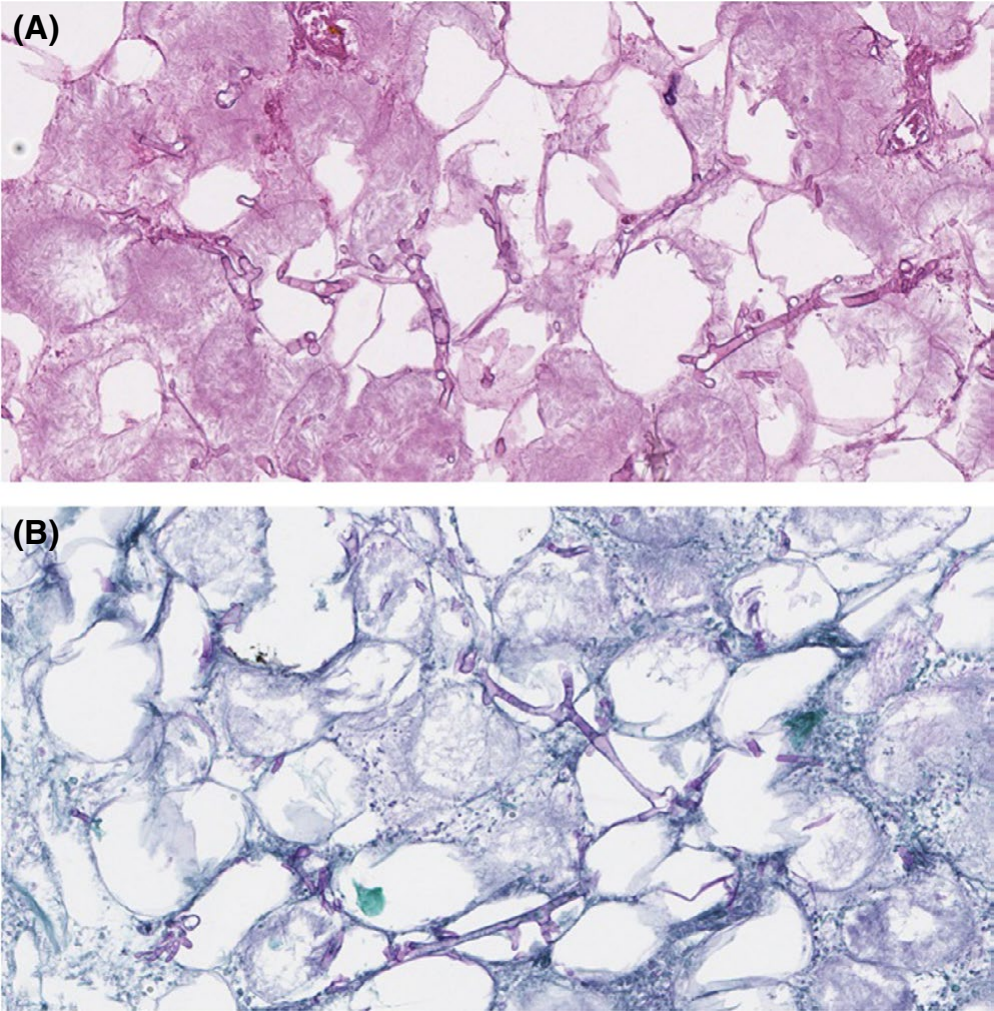
A, H&E with extensive fat necrosis and fungal elements of variable thickness with septate and acute angle branching (20×). B, PAS stain highlighting numerous fungal hyphae in magenta within areas of fat necrosis (20×)

A middle‐aged man with a history only remarkable for alcohol abuse presented as an acute trauma after his snowmobile collided into a tree at high speed. The patient had numerous fractures of his bilateral lower extremities and spine, as well as a Lefort II and mandible fracture requiring maxillary and mandibular plating. Following hemodynamic stabilization, his laboratory tests revealed a white blood cell count (WBC) of 4100/mcL, hematocrit of 25%, platelet count of 131 000/mcL, and glucose of 96 mg/dL. On hospital day 4, the patient became febrile with newly elevated WBC of 13 200/mcL and an increasing vasopressor requirement. Though there was no clear source of infection, he was started on empiric, broad‐spectrum antibiotics. The patient continued to be febrile throughout his hospitalization, and he developed an increasing leukocytosis. Repeat routine blood cultures were consistently negative. On day 15, the patient developed right‐sided facial swelling with findings suggestive of a fluid collection along the right maxilla on CT imaging. His WBC was severely elevated at 43 000/mcL, and his glucose level was 222 mg/dL. These findings were initially attributed to a postoperative infection. An incision and drainage was attempted which yielded no fluid, and the patient was maintained on empiric antibiotics. On day 18, the patient was noted to have a rapidly expanding necrotic eschar over his right temple and malar cheek, and his white count remained elevated at 35 000/mcL. The primary team initially attributed these findings to frostbite, but unfortunately no photographs were available from the lesion at that time. Dermatology was consulted on day 22 as the eschar continued to grow, and subsequent examination revealed a large atrophic, stellate violaceous‐to‐necrotic plaque over his right temporal and malar cheek with areas of underlying edema and adjacent necrotic papules with periorbital edema and jaundice (Figure 1A). Of note, the distal nose and helix of the right ear were spared, which placed frostbite lower on the differential diagnosis and raised concern for an invasive fungal infection. Two punch biopsies of the right cheek were taken for histopathologic analysis and culture. Follow‐up examination the next day revealed the rapid expansion of the necrotic plaque (Figure 1B). Histopathology revealed extensive tissue necrosis with the involvement of the subcutaneous fat with abundant hyphae (Figure 2A) that were highlighted by Periodic acid‐Schiff stain (Figure 2B). The patient underwent surgical debridement of necrotic tissue, and treatment with amphotericin B was initiated. However, given the combination of traumatic brain injury, multiple fractures, and angioinvasive fungal infection, the patient was placed on hospice and eventually passed.

## 3 DISCUSSION

Often thought of as a disease exclusive to the immunocompromised, the cases reported of immunocompetent angioinvasive fungal infections point toward trauma as a precipitating factor.^3^, ^4^, ^5^ Rhizopus species is known to be ubiquitous in soil and decaying vegetation.^6^ In our present case, we suspect inoculation occurred from the patient's impact with a tree, although no data are available to confirm the presence of fungal spores on the native tree population in our rural environment. This case serves to highlight the often unreported, technical complexity which such a case of traumatic inoculation in an immunocompetent host may present fractures requiring numerous surgeries and consulting physician, hardware placement, and an unidentified infection which may evade routine cultures requiring empirical antibiotic treatment. With multiple surgeries and consulting physicians, the diagnosis of invasive fungal infections is often elusive. In the setting of trauma, even in an immunocompetent host, invasive fungal infection should be added and remain on the differential, particular in the setting of unexplained leukocytosis as in this case.

Often, the first clue of an invasive fungal infection is the manifestation of cutaneous signs. This is where the role of the dermatology becomes a necessity in these cases, and their role has yet to be defined in the literature. Dermatology has the clinical training and diagnostic tools to aid in the rapid diagnosis and treatment of a rare presentation of immunocompetent mucormycosis. Early signs of cutaneous mucormycosis include pustules, blisters, nodules, necrotic ulcerations, ecthyma gangrenosum‐like lesions, and necrotizing cellulitis.^2^ This differs from frostbite which most commonly presents as deep red or blue/gray discoloration and necrosis on the nose and ears,^7^ not sparing the helix in the described case, a distinction that would be difficult for any clinician. Of further note, cutaneous mucormycosis is difficult to identify early in its course and is rarely diagnosed with cultures. It almost always requires a biopsy with histopathology demonstrating evidence of fungal infection, such as the present case.^3^ Dermatology can perform rapid frozen tissue section, which can aid in same day recognition and treatment, but even so can still result in misdiagnosis. Frozen tissue section in this case originally yielded acute septate consistent with Aspergillus, which was highest on our differential, but repeat tissue section showed ninety‐degree branching and culture grew back Rhizopus species. Had the mistake been made to start the patient on voriconazole, he would have had several days to weeks of improper antifungal regimen while awaiting culture results. This points to a future area of research for us which is to determine the specificity of fungal infection pathogen diagnosis using rapid frozen tissue section. Given the high fatality of invasive mucormycosis, early recognition and treatment of the disease is essential. This can often be complicated by the setting of trauma, but dermatology can recognize the earliest cutaneous signs and assist in the treatment of this highly evasive pathogen.

## CONFLICT OF INTEREST

None reported.

## AUTHOR CONTRIBUTIONS

BJS and DJB: had full access to all of the data in the study and take responsibility for the integrity of the data and the accuracy of the data analysis. BJS, KAZ, and DJB: conceived and designed the study. BJS and DJB: acquired, analyzed, and interpreted the data. DJB and BJS: drafted the manuscript. KAZ, SY, BJS, DJB, and CB: critically revised the manuscript for important intellectual content. KAZ, SY: provided administrative, technical, or material support, and supervised the study. N/A: involved in statistical analysis and obtained funding.
